# The Impact of Asymptomatic Human Immunodeficiency Virus-Positive Disease Status on Inpatient Complications Following Spine Surgery: A Propensity Score-Matched Analysis

**DOI:** 10.3390/jcm12041458

**Published:** 2023-02-12

**Authors:** Neil V. Shah, Matthew J. Lettieri, Samuel Gedailovich, David Kim, Madhu Oad, Ryne J. Veenema, Adam J. Wolfert, George A. Beyer, Hanbin Wang, Ravi S. Nunna, Douglas A. Hollern, Renaud Lafage, Vincent Challier, Andrew A. Merola, Peter G. Passias, Frank J. Schwab, Virginie Lafage, Carl B. Paulino, Bassel G. Diebo

**Affiliations:** 1 Department of Orthopedic Surgery and Rehabilitation Medicine, State University of New York (SUNY) Downstate Health Sciences University, Brooklyn, NY 11203, USA; 2 Department of Radiology, Nassau University Medical Center, Hempstead, NY 11550, USA; 3 Department of Neurological Surgery, University of Missouri Columbia, Columbia, MO 65211, USA; 4 Department of Orthopaedic Surgery, Northwell Health Lenox Hill Hospital, New York, NY 10075, USA; 5 Spine Unit 1, Orthopedic Surgery Department, Bordeaux University Hospital, 33400 Bordeaux, France; 6 Department of Orthopaedic Surgery, NewYork-Presbyterian Brooklyn Methodist Hospital, Brooklyn, NY 11215, USA; 7 Department of Orthopaedic Surgery, NYU Langone Orthopedic Hospital, New York, NY 10021, USA; 8 Department of Orthopaedic Surgery, Warren Alpert School of Medicine, Brown University, East Providence, RI 02912, USA

**Keywords:** anterior cervical discectomy and fusion, lumbar fusion, thoracolumbar fusion, asymptomatic HIV, complications

## Abstract

In the United States, nearly 1.2 million people > 12 years old have human immunodeficiency virus (HIV), which is associated with postoperative complications following orthopedic procedures. Little is known about how asymptomatic HIV (AHIV) patients fare postoperatively. This study compares complications after common spine surgeries between patients with and without AHIV. The Nationwide Inpatient Sample (NIS) was retrospectively reviewed from 2005–2013, identifying patients aged > 18 years who underwent 2–3-level anterior cervical discectomy and fusion (ACDF), ≥4-level thoracolumbar fusion (TLF), or 2–3-level lumbar fusion (LF). Patients with AHIV and without HIV were 1:1 propensity score-matched. Univariate analysis and multivariable binary logistic regression were performed to assess associations between HIV status and outcomes by cohort. 2–3-level ACDF (*n* = 594 total patients) and ≥4-level TLF (*n* = 86 total patients) cohorts demonstrated comparable length of stay (LOS), rates of wound-related, implant-related, medical, surgical, and overall complications between AHIV and controls. 2–3-level LF (*n* = 570 total patients) cohorts had comparable LOS, implant-related, medical, surgical, and overall complications. AHIV patients experienced higher postoperative respiratory complications (4.3% vs. 0.4%,). AHIV was not associated with higher risks of medical, surgical, or overall inpatient postoperative complications following most spine surgical procedures. The results suggest the postoperative course may be improved in patients with baseline control of HIV infection.

## 1. Introduction

Nearly 40 million people worldwide currently live with human immunodeficiency virus (HIV), with about 1.2 million individuals aged > 12 years living in the United States (US) [1,2]. HIV is a retrovirus that causes a decline in CD4+ T cell number and function over time [3]. While the 1980s showcased the devastating introduction of this deadly infection into the US, HIV has become a more manageable condition since the inception of antiretroviral therapy (ART) regimens [4].

These advances in HIV treatment have led to marked improvements in life expectancy in this population with an estimated global increase of 14.4 million life-years in adults from 1995 to 2009 [5,6]. Samji et al. [7] noted that an HIV-positive young adult living in the US or Canada who is treated with ART can be expected to live well into their early 70s. In an aging population, there is an increase in conditions such as osteoarthritis, adult spinal deformity, and degenerative disc disease, with operative management available as a treatment option [8,9,10].

In the absence of acute HIV-related illnesses or the progression towards acquired immunodeficiency syndrome (AIDS), patients experience asymptomatic HIV (AHIV). Patients can remain in this chronic, asymptomatic state for 10 years or longer [11]. Studies have shown that early initiation of ART during this asymptomatic phase can delay the progression from HIV to AIDS as well as prevent complications associated with HIV including death [12].

Extensive research has demonstrated an increased risk of postoperative complications among those with symptomatic HIV undergoing orthopedic procedures [13,14,15,16,17]. However, the literature investigating the AHIV population is scant. With the success of HIV treatment and its ability to increase life expectancy, the asymptomatic population of HIV patients will continually age, and a presumably higher proportion of affected patients will experience and develop degenerative musculoskeletal pathologies.

Many of these degenerative disease processes impact the spine, including cervical radiculopathy and myelopathy. In many cases, anterior cervical discectomy and fusion (ACDF) has become the standard method of treatment, with most outcomes having satisfactory results. However, hematoma and recurrent laryngeal nerve palsy are notable for being possible complications [18]. Conditions such as degenerative disc disease also prove to be very common throughout the US and can affect up to two-thirds of adults throughout their lifetime [19]. Lumbar fusion is often accepted as an effective technique in the treatment of such disease, with common complications including dural tear and nerve root injury [20]. In the case of adult spinal deformity (ASD), although surgical techniques, such as thoracolumbar fusion, have improved significantly over the past years, complications such as implant failure remain common and may be a significant source of patient morbidity [21].

Taking into consideration that symptomatic HIV patients are at increased risk of postoperative complications following orthopedic surgery [13,14,15,16,17], the goal of this study was to determine if risks of adverse postoperative outcomes do not increase in patients with AHIV. This study compared the postoperative outcomes of patients with and without AHIV who underwent spine surgery. This study investigated postoperative complications of those undergoing ACDF for cervical radiculopathy or myelopathy, thoracolumbar fusion for ASD, and lumbar fusion for degenerative disc disease.

## 2. Materials and Methods

### 2.1. Data Source, Patient Selection and Inclusion Criteria

The Nationwide Inpatient Sample (NIS) was retrospectively reviewed from 2005–2013. Using the International Classification of Diseases, Ninth Revision, Clinical Modification (ICD-9-CM) codes, adult patients (>18 years of age) were selected if they underwent spinal fusion for an included diagnosis. This study was deemed exempt from review by our institutional review board (Study ID: 1847009-2).

Patients were divided into three cohorts based on the spinal region fused. The first cohort consisted of patients with cervical radiculopathy (72.10, 72.20, 72.24, 722.81, 722.91) or myelopathy (72.11, 722.71) who underwent 2–3-level ACDF (81.02, 81.03, 81.32, 81.33). The second cohort consisted of patients with ASD, identified as those with idiopathic scoliosis (737.30, 737.32) or degenerative disc disease (737.10, 737.20, 722.52, 722.51, 724.02, 721.3, 738.4, 722.10, 756.12, 722.73, 721.42, 724.01, 721.2, 722.72, 721.41, 722.11, 724.03, 756.11) who underwent ≥4-level thoracolumbar fusion (81.63, 81.64). The third cohort consisted of patients with degenerative disc disease who underwent 2–3-level lumbar fusion (81.62). Patients in each procedural cohort were then stratified based on the presence or absence of AHIV (ICD9 CM code V08), indicating if a patient is positive for HIV infection but no longer has detectable levels.

### 2.2. Exclusion Criteria

Patients with any other form of HIV, history or presence of an HIV-related illness, or AIDS were excluded. Patients were excluded if they had osteomyelitis, pathologic fractures, traumatic fractures, or any cancer.

### 2.3. Statistical Analysis

Within each procedural cohort, patients with and without AHIV were 1:1 propensity score-matched by age, sex, race, and insurance status. The outcome measures analyzed included length of stay (LOS), total hospital charges, medical complications, surgical complications, and mortality. The NIS tracks outcomes during the inpatient stay following the index surgery through discharge from the hospital. Univariate analysis was used to compare demographics and outcomes measures in each cohort between patients with and without AHIV. Chi-squared analysis was used to compare categorical variables and two-tailed independent t-tests were used to compare continuous variables. Multivariable logistical regression was used to identify independent predictors of adverse outcomes among each surgical cohort, with AHIV status, age, sex, and race as covariates. Statistical significance was set at *p* < 0.05. All analyses were completed using SPSS Statistics version 24.0 (IBM Corp., Armonk, NY, USA).

## 3. Results

### 3.1. 2–3-Level Anterior Cervical Discectomy and Fusion

A total of 594 patients who underwent 2–3-level ACDF were included. Of the total, 297 patients had a diagnosis of AHIV and were matched to 297 who did not. There was no significant difference between the AHIV group and the non-HIV group in age (49.2 vs. 49.9, respectively; *p* = 0.373), sex (both cohorts 75.8% male; *p* = 1.000), race (*p* = 0.671), and insurance status (*p* = 0.865). Patients with AHIV and non-HIV patients had comparable total cost of hospitalization (USD 54,275.91 vs. USD 48,286.99, respectively; *p* = 0.093) and LOS (2.1 vs. 1.8 days, respectively; *p* = 0.138) (Table 1).

The overall complication rate was comparable for patients with AHIV and non-HIV patients (3.1% vs. 3.0%, respectively; *p* = 0.912) as well as overall (2.6% vs. 2.7%, respectively; *p* = 0.794) and individual medical complications, including acute renal failure (1.0% vs. 0.3%, respectively; *p* = 0.316), deep vein thrombosis (0.3% vs. 0%, respectively; *p* = 0.317), pneumonia (0% vs. 0.7%, respectively; *p* = 0.157), and respiratory complications (0% vs. 0.3%, respectively; *p* = 0.317). Patients with AHIV and non-HIV patients also experienced similar rates of overall (0.7% vs. 0.3%, respectively; *p* = 0.563) and individual surgical complications, including wound infections (0.3% vs. 0%, respectively; *p* = 0.317) and implant-related complications (0.3% vs. 0.3%, respectively; *p* = 1.000). Neither group experienced mortality (Table 2).

Regression analysis revealed that AHIV was not an independent predictor of overall (OR = 0.8, CI: 0.3–2.2; *p* = 0.698), medical (OR = 0.7, CI: 0.2–2.1; *p* = 0.705), or surgical complications (OR = 2.1, CI: 0.2–29.4; *p* = 0.576).

### 3.2. ≥4-Level Thoracolumbar Fusion

Eighty-six patients who underwent ≥4-level thoracolumbar fusion were included, with 43 AHIV patients and 43 patients without AHIV. No significant differences were observed between patients with AHIV and non-HIV patients in mean age (53.3 vs. 51.1 years, respectively; *p* = 0.43), sex (86.0% vs. 90.7% male, respectively; *p* = 0.507), race (*p* = 0.880), and insurance status (*p* = 0.886). Cost of hospitalization (USD 152,376.44 vs. USD137,630.10, respectively; *p* = 0.434) and hospital length of stay (7.0 vs. 4.5 days, respectively; *p* = 0.082) and were also similar between the two cohorts (Table 3).

Patients with AHIV and non-HIV patients experienced comparable overall complications (41.9% vs. 30.2%, respectively; *p* = 0.189). There was no significant difference in overall (34.9% vs. 20.9%, respectively; *p* = 0.149) or individual medical complications, including acute renal failure (2.3% vs. 2.3%, respectively; *p* = 1.00), anemia (23.3% vs. 14.0%, respectively; *p* = 0.268), acute respiratory distress syndrome (2.3% vs. 0%, respectively; *p* = 0.314), cardiac complications (4.7% vs. 0%, respectively; *p* = 0.152), deep vein thrombosis (2.3% vs. 2.3%; *p* = 1.00), and infection (2.3% vs. 0%, respectively; *p* = 0.314. Overall (7.0% vs. 9.3%, respectively; *p* = 0.485) and individual surgical complications did not differ significantly between patients with AHIV and non-HIV patients, including wound disruption (2.3% vs. 0%, respectively; *p* = 0.314), implant infection (2.3% vs. 0%, respectively; *p* = 0.314), and implant-related complications (0% vs. 7%, respectively; *p* = 0.152) (Table 4).

Regression analysis revealed that AHIV was not an independent predictor of overall (OR = 1.5, CI: 0.6–4.1; *p* = 0.403), medical (OR = 1.9, CI: 0.6–5.5; *p* = 0.247), or surgical complications (OR = 1.2, CI: 0.2–7.1; *p* = 0.824).

### 3.3. 2–3-Level Lumbar Fusion

A total of 570 patients who underwent 2–3-level lumbar fusion were included, comprising 285 patients with AHIV and 285 patients without AHIV. There was no significant difference observed between patients with AHIV and non-HIV patients in age (49.8 vs. 49.7 years, respectively; *p* = 0.903), sex (72.3% vs. 71.6% male, respectively; *p* = 0.852), race (*p* = 0.395), or insurance status (*p* = 0.875). There was also no significant difference in cost of hospitalization (USD 97,194.67 vs. USD 89,877.74, respectively; *p* = 0.155) or mean LOS (4.0 vs. 3.8 days, respectively; *p* = 0.585) between patients with AHIV and non-HIV (Table 5).

Overall medical complications were comparable between patients with AHIV and non-HIV patients (15.8% vs. 13.7%, respectively; *p* = 0.478). Of note, patients with AHIV experienced higher rates overall respiratory complications compared to non-HIV patients (4.3% vs. 0.4%, respectively; *p* < 0.033). Other individual medical complications were similar, including acute renal failure (1.4% vs. 1.1%, respectively; *p* = 0.704), anemia (8.4% vs. 7.7%, respectively; *p* = 0.758), deep vein thrombosis (0.7% vs. 1.4%, respectively; *p* = 0.412), hematoma (1.4% vs. 0.4%, respectively; *p* = 1.000), and infection (0.4% vs. 0.4%; *p* = 1.000). The overall rate of surgical complications (3.2% vs. 3.2%; *p* = 1.000) was identical for patients with AHIV and the control group. Individual surgical complications were also comparable between AHIV and non-HIV patients, including wound disruption (0.7% vs. 0%, respectively; *p* = 0.157), implant infection (0% vs. 0.4%, respectively; *p* = 0.317), and implant-related complications (0.4% vs. 1.1%, respectively; *p* = 0.316). Both patients with AHIV and non-HIV patients experienced similar rates of overall complications (18.6% vs. 16.1%, respectively; *p* = 0.439) and mortality (0.40% vs. 0.00%, respectively; *p* > 0.05) (Table 6).

Regression analysis revealed that AHIV was not an independent predictor of overall (OR = 1.1, CI: 0.7–1.7; *p* = 0.716), medical (OR = 1.1, CI: 0.7–1.8; *p* = 0.669), or surgical (OR = 0.8, CI: 0.3–2.3; *p* = 0.716) complications following 2–3-level lumbar fusion.

## 4. Discussion

With the advent of ART regimens and its positive effect on life expectancy, the last several decades have witnessed a rise in the number of people living with HIV [2]. Life expectancies have been projected to be as high as 78 years in Europe and North America, with ART maintaining HIV-positive patients in a chronic, asymptomatic stage [22]. However, increased life expectancy has associated age-related orthopedic conditions, such as osteoarthritis, adult spinal deformity, and degenerative disc disease, with consequent potential need for surgical management [9,10,23]. While current research shows increased complication rates in symptomatic HIV patients undergoing orthopedic procedures when compared to the general population [13,14,15,16,17], the focus of the current study was on asymptomatic HIV patients and the impact of AHIV on outcomes after ACDF, thoracolumbar fusion, and short-segment lumbar fusion.

Across all three surgical procedures, patients with and without AHIV had a similar rate of complications to patients without AHIV. These results support the preliminary findings in the study performed by Young et al. [24], which investigated elective spinal surgery in asymptomatic HIV patients. While their sample was limited to 11 patients, it included procedures such as ACDF, LF, and discectomy. Their results showed only 2 out of 11 patients incurred postoperative complications. A retrospective cohort study of the PearlDiver Patient Records Database found that AHIV patients who underwent lumbar fusion were not at greater risk of major complications except for respiratory complications within 90 days compared to non-HIV patients [25]. The authors of this study have suggested higher rates of smoking in patients with HIV as an explanation for the increased rates of respiratory complications [25]. Our results reinforce and build on these findings with a retrospective analysis of a larger cohort of AHIV patients being analyzed following three different spinal surgeries.

The results of this study also corroborate research carried out in other orthopedic disciplines. For example, Bahebeck et al. [26] found comparable infection rates between patients with AHIV and patients without HIV in a variety of orthopedic procedures in which implants were used, including intramedullary nailing, plating, pinning, or arthroplasty, if on an ART regimen and antibiotics. In addition, Hoekman et al. [27] found comparable postsurgical infection rates between AHIV patients and HIV-negative patients undergoing open reduction and internal fixation (0% and 5%, respectively), while symptomatic HIV patients had a greater infection rate of 23%. These findings can help orthopedic surgeons optimize patients preoperatively and underscore the importance of maintaining HIV-positive patients in an asymptomatic state.

On the other hand, O’Brien et al. [28] found higher postoperative infection rates in patients with AHIV patients compared to patients without HIV undergoing tibial fracture fixation. Four out of the total 15 patients had postoperative infections, with 3 out of 4 attributed to the AHIV cohort. Furthermore, Paiement et al. [29] found that trauma patients with AHIV had greater rates of postoperative infections compared with HIV-negative trauma patients (16.7% and 5.4%, respectively), especially among those with open fractures, with an even greater difference of 55.6% vs. 11.3%, respectively. However, both of these studies have a smaller sample size and were performed nearly two decades ago. Since that time, there have been substantial advancements in HIV treatment, which has the ability to impact outcomes [30]. Further research to quantify the impact of HIV treatment in orthopedic surgery is warranted [31].

This study is not without limitations. This study retrospectively reviewed the NIS database, in which only inpatient data without identifiers for longitudinal follow-up were provided. A second limitation was the ICD-9 coding used to identify complications and HIV status. The true CD4 count of patients is not known; thus, there is a strong reliance on coding to appropriately identify HIV and AHIV patients. Additionally, there was a lack of data on other risk factors for postoperative infections, such as intraoperative bleeding and duration of the surgery. As an administrative database, there is also inherent variability in the coding and inclusion of different diagnoses and procedures. Another limitation was the small patient population, which ranged from 594 in our ACDF cohort to 86 patients in our TLSF cohort. It is possible that the cohorts are potentially smaller than they may truly be, as identification of these patients required the ICD-9 code for AHIV to be included in the patient’s record. Yet, though small, this study represents the most comprehensive analysis of AHIV patients and the impacts on objective medical and surgical outcomes following three common spine surgical procedures.

## 5. Conclusions

This study investigated the rate of complications among those with AHIV compared to those without HIV following ACDF, thoracolumbar fusion for ASD, and short-segment lumbar fusion for degenerative disc disease. AHIV was not associated with an increased risks of overall, medical, or surgical complications among patients undergoing these spinal procedures. Overall respiratory complications were observed to be higher among AHIV patients when compared to non-HIV (4.3% vs. 0.4%) patients following short-segment lumbar fusion, supporting previously reported findings. These results suggest that baseline asymptomatic HIV status lacks association with incidence of overall adverse postoperative complications following surgery. It is unclear whether medical optimization of HIV-positive patients is impacted by gaining or maintaining asymptomatic status. These results can help spine surgeons counsel HIV-positive patients on treatment risks and expectations as part of their preoperative discussions when considering spine surgery.

## Figures and Tables

**Table 1:** Patient demographic data for asymptomatic HIV (AHIV) and non-HIV patients undergoing 2–3-level anterior cervical discectomy and fusion for cervical radiculopathy or myelopathy.

	AHIV	Non-HIV	*p*-Value
*n*	297	297	-
Age (years)	49.2	49.9	0.373
Sex			
Males	75.8%	75.8%	1.000
Females	24.2%	24.2%	
Race			
White	59.9%	76.1%	0.671
Black	30.3%	7.4%	
Hispanic	5.7%	5.7%	
Asian	0%	2.7%	
Native American	0.3%	1.3%	
Other	3.7%	6.7%	
Insurance Status			
Medicare	36.0%	33.3%	0.865
Medicaid	17.5%	10.8%	
Private Insurance	38.0%	52.5%	
Self-Pay	2.4%	0.3%	
No Charge	0.7%	0%	
Other	5.4%	3.0%	
Total Charges	USD 54,275.91	USD 48,286.99	0.093
Hospital Length of Stay	2.1 days	1.8 days	0.138

**Table 2:** Medical and surgical complications in asymptomatic HIV (AHIV) and non-HIV patients undergoing 2–3-level anterior cervical discectomy and fusion for cervical radiculopathy or myelopathy.

	AHIV	Non-HIV	*p*-Value
Medical Complications	2.6%	2.7%	0.794
Acute renal failure	1.0%	0.3%	0.316
Altered mental status	0%	0.3%	0.317
Anemia	0.7%	0.3%	0.563
Deep vein thrombosis	0.3%	0%	0.317
Hematoma	0.3%	1.0%	0.316
Pneumonia	0%	0.7%	0.157
Acute respiratory distress syndrome	0%	0.3%	0.317
Vascular injury	0%	0.3%	0.317
Peripheral vascular disease	0.3%	0%	0.317
Surgical Complications	0.7%	0.3%	0.563
Wound infection	0.3%	0%	0.317
Implant-related complications	0.3%	0.3%	1.000
Overall Complications	3.1%	3.0%	0.912

**Table 3:** Patient demographic data for asymptomatic HIV (AHIV) and non-HIV patients undergoing ≥ 4-level thoracolumbar fusion for adult spinal deformity.

	AHIV	Non-HIV	*p*-Value
*n*	43	43	-
Age (years)	53.3	51.1	0.430
Sex			
Males	86.0%	90.7%	0.507
Females	14.0%	9.3%	
Race			
White	62.8%	72.1%	0.880
Black	25.2%	9.3%	
Hispanic	4.7%	9.3%	
Asian	0%	0%	
Native American	0%	0%	
Other	7.0%	9.3%	
Insurance Status			
Medicare	39.5%	39.5%	0.886
Medicaid	11.6%	11.6%	
Private Insurance	34.9%	39.5%	
Self-Pay	4.7%	0%	
No Charge	0%	0%	
Other	9.3%	9.3%	
Total Charges	USD 152,376.44	USD 137,630.10	0.434
Hospital Length of Stay	7.0 days	4.5 days	0.082

**Table 4:** Medical and surgical complications in asymptomatic HIV (AHIV) and non-HIV patients undergoing ≥ 4-level thoracolumbar fusion for adult spinal deformity.

	AHIV	Non-HIV	*p*-Value
Medical Complications	34.9%	20.9%	0.149
Acute renal failure	2.3%	2.3%	-
Anemia	23.3%	14.0%	0.268
Acute respiratory distress syndrome	2.3%	0%	0.314
Cardiac complications	4.7%	0%	0.152
Digestive complications	0%	2.3%	0.314
Deep vein thrombosis	2.3%	2.3%	-
Hematoma	0%	0%	-
Infection	2.3%	0%	0.314
Nervous system complications	0%	2.3%	0.314
Pulmonary embolism	0%	0%	-
Pneumonia	0%	0%	-
Surgical Complications	7.0%	9.3%	0.485
Wound disruption	2.3%	0%	0.314
Wound infection	2.3%	0%	0.314
Implant infection	2.3%	0%	0.314
Implant-related complications	0%	7.0%	0.152
Dural tear	0%	2.3%	0.314
Overall Complications	41.9%	30.2%	0.189

**Table 5:** Patient demographic data for asymptomatic HIV (AHIV) and non-HIV patients undergoing 2–3-level lumbar fusion for degenerative disc disease.

	AHIV	Non-HIV	*p*-Value
*n*	285	285	-
Age (years)	49.8	49.7	0.903
Sex			
Males	72.3%	71.6%	0.852
Females	27.7%	28.4%	
Race			
White	57.5%	76.8%	0.395
Black	29.8%	7.0%	
Hispanic	8.1%	8.4%	
Asian	0.7%	1.1%	
Native American	0.7%	2.1%	
Other	3.2%	4.6%	
Insurance Status			
Medicare	31.9%	33.7%	0.875
Medicaid	17.9%	6.7%	
Private Insurance	40.4%	54.0%	
Self-Pay	1.4%	0.4%	
No Charge	1.0%	0%	
Other	7.4%	5.3%	
Total Charges	USD 97,194.67	USD 89,877.74	0.155
Hospital Length of Stay	3.96 days	3.8 days	0.585

**Table 6:** Medical and surgical complications in asymptomatic HIV (AHIV) and non-HIV patients undergoing 2–3-level lumbar fusion for degenerative disc disease.

	AHIV	Non-HIV	*p*-Value
Medical Complications	15.8%	13.7%	0.478
Acute renal failure	1.4%	1.1%	0.704
Acute myocardial infarction	0%	0.4%	0.317
Altered mental status	0.4%	0.4%	-
Anemia	8.4%	7.7%	0.758
Acute respiratory distress syndrome	0.4%	0.4%	-
Cardiac complications	0%	0.4%	0.317
Digestive complications	1.4%	0.4%	0.178
Deep vein thrombosis	0.7%	1.4%	0.412
Hematoma	1.4%	0.4%	0.178
Infection	0.4%	0.4%	-
Pulmonary embolism	0%	0.7%	0.157
Pneumonia	1.1%	1.1%	-
**Overall respiratory complications**	**4.3%**	**0.4%**	**<0.033**
Peripheral vascular disease	0%	0.4%	0.317
Urinary	1.1%	1.1%	-
Surgical Complications	3.2%	3.2%	-
Wound disruption	0.7%	0%	0.157
Implant infection	0%	0.4%	0.317
Implant-related complications	0.4%	1.1%	0.316
Overall Complications	18.6%	16.1%	0.439

## Data Availability

Publicly available datasets were analyzed in this study.
